# Brain structural deficits and working memory fMRI dysfunction in young adults who were diagnosed with ADHD in adolescence

**DOI:** 10.1007/s00787-015-0755-8

**Published:** 2015-08-26

**Authors:** Andres Roman-Urrestarazu, Päivi Lindholm, Irma Moilanen, Vesa Kiviniemi, Jouko Miettunen, Erika Jääskeläinen, Pirjo Mäki, Tuula Hurtig, Hanna Ebeling, Jennifer H. Barnett, Juha Nikkinen, John Suckling, Peter B. Jones, Juha Veijola, Graham K. Murray

**Affiliations:** Department of Psychiatry, University of Cambridge, Box 189 Addenbrooke’s Hospital, Cambridge, CB2 0QQ UK; Department of Child Psychiatry, Institute of Clinical Medicine, University of Oulu and Oulu University Hospital, Oulu, Finland; Department of Diagnostic Radiology, Institute of Diagnostics, Oulu University Hospital, Oulu, Finland; Department of Psychiatry, Institute of Clinical Medicine, University of Oulu and Oulu University Hospital, Oulu, Finland; Department of Public Health Sciences and General Practice, Institute of Health Sciences, University of Oulu, Oulu, Finland; Medical Research Center Oulu, University of Oulu and Oulu University Hospital, Oulu, Finland; Department of Oncology and Radiotherapy, Oulu University Hospital, Oulu, Finland; NIHR Cambridge Biomedical Research Centre, Cambridge, UK; Behavioural and Clinical Neuroscience Institute, University of Cambridge, Cambridge, UK

**Keywords:** MRI, Memory, ADHD, Hyperkinetic, VBM, Neuroanatomy

## Abstract

When adolescents with ADHD enter adulthood, some no longer meet disorder diagnostic criteria but it is unknown if biological and cognitive abnorma lities persist. We tested the hypothesis that people diagnosed with ADHD during adolescence present residual brain abnormalities both in brain structure and in working memory brain function. 83 young adults (aged 20–24 years) from the Northern Finland 1986 Birth Cohort were classified as diagnosed with ADHD in adolescence (adolescence ADHD, *n* = 49) or a control group (*n* = 34). Only one patient had received medication for ADHD. T1-weighted brain scans were acquired and processed in a voxel-based analysis using permutation-based statistics. A sub-sample of both groups (ADHD, *n* = 21; controls *n* = 23) also performed a Sternberg working memory task whilst acquiring fMRI data. Areas of structural difference were used as a region of interest to evaluate the implications that structural abnormalities found in the ADHD group might have on working memory function. There was lower grey matter volume bilaterally in adolescence ADHD participants in the caudate (*p* < 0.05 FWE corrected across the whole brain) at age 20–24. Working memory was poorer in adolescence ADHD participants, with associated failure to show normal load-dependent caudate activation. Young adults diagnosed with ADHD in adolescence have structural and functional deficits in the caudate associated with abnormal working memory function. These findings are not secondary to stimulant treatment, and emphasise the importance of taking a wider perspective on ADHD outcomes than simply whether or not a particular patient meets diagnostic criteria at any given point in time.

## Introduction

Attention deficit/hyperactivity disorder (ADHD) [1–4] was initially thought to abate in adolescence but increasing evidence indicates that ADHD frequently persists through to adulthood [3, 4]. Of those diagnosed during childhood, about 30–60 % show symptoms during adulthood [3, 5, 6]. It is because of its associated morbidity and disability across the lifespan that ADHD has come to be a major clinical and public health concern [7].

Although the precise neural and pathophysiological substrates of ADHD remain unknown, there is evidence that indicates abnormalities in fronto-striatal and limbic pathways [8–16]. However, inconsistency in the existing research evidence, combined with the concern that most evidence is potentially confounded by the effects of medication, means that the pathology of the disorder remains uncertain [8, 14, 17]. To some extent, differences in referral and diagnostic practices in different clinical and research centres also mean that study recruitment biases may also contribute to the heterogeneity of findings in the condition.

A variety of different outcome measures are important when considering the long-term prognosis of children diagnosed with ADHD. Relevant and important follow-up outcomes include whether or not individuals continue to meet diagnostic criteria for ADHD, the number and severity of ADHD symptoms they retain, the presence of comorbid psychiatric illness, social and occupational outcomes, and neurological and neuropsychological outcomes, including brain structure and function and associated cognitive performance or deficit [3, 17–20]. Adults with ADHD have a variety of neurocognitive deficits of which working memory is one of the more significant, and has been proposed to be a core feature of the disorder [21]. Whilst a number of studies have examined prognosis in terms of psychiatric symptoms’ outcomes, far fewer studies have examined outcomes in terms of brain structure and function, in spite of the possibility that such measures may relate closely to social and occupational success [17, 22, 23].

This study aims to investigate young adult brain structural and functional (in terms of working memory) outcomes of people diagnosed with ADHD during adolescence using a general population-based methodology. The general population-based nature of our study helps us examine ADHD with less, or possibly different, selection biases, compared to the average study of ADHD. Clinical practice in Northern Finland at the time our participants were diagnosed with ADHD did not rely on stimulant medication in the treatment; this enabled us to avoid the potentially confounding effects of medication from the results, as only one subject was taking ADHD medication at the time of the scans and no other participants had been treated with stimulants previously. We hypothesised that there would be residual brain structural, brain functional and working memory deficits in young adults who had been diagnosed with ADHD in adolescence.

## Methods

### Participant selection process

Fifty two young adults who had been diagnosed with DSM-IV ADHD at age 16–17 (henceforth “adolescence ADHD”) and 34 young adult controls without ADHD gave written consent and took part in the study, which was approved by the ethical committee of the Northern Ostrobothnia Hospital District, Finland. All participants were members of The Northern Finland 1986 Birth Cohort (NFBC1986) and were all aged 20–24 at the time of the study. We have previously published details of how adolescents with ADHD were identified within the birth cohort in a general population-based approach [24–28], and we summarise the details here. The birth cohort population from whom the participants were selected was composed of children with an expected date of birth between July 1st, 1985 and June 30th, 1986, in the two northernmost provinces of Finland (Oulu and Lapland). This population-based birth cohort included 99 % of all births in the area at that time and consisted of 9479 children, of whom 9432 were live-born [29] (http://kelo.oulu.fi/NFBC). Parents of cohort members were asked to complete the Strengths and Weaknesses of ADHD Symptoms and Normal Behaviours (SWAN) questionnaire [30] at age 15–16 that measured problems of attention and hyperactivity. Among the 6622 respondents to the survey, a subset of 457 possible cases and general population controls were identified based on their questionnaire scores, and these adolescents were evaluated for ADHD during 2002–2003 in a clinical evaluation, including a structured interview (Schedule for Affective Disorders and Schizophrenia for School-Age Children-Present and Lifetime version, K-SADS-PL) [24, 26–28, 31]. 105 cases were diagnosed having current, definite ADHD according to DSM-IV at the age of 16–18. A sample of healthy control participants also from NFBC1986 (therefore matched to ADHD participants in age, place of birth and many environmental influences) without current or previous ADHD at age 16–18 were also identified prospectively in adolescence and invited to take part in the young adult study.

Cohort members with ADHD at age 16–18 (adolescence ADHD), and a sample of the controls without ADHD identified at age 16–18, were, several years later, invited to participate in the current study performed in 2007–2010 at Oulu University Hospital, Finland. Of the 105 adolescence ADHD individuals, and therefore eligible to participate in this study, 52 (50 %) were scanned, along with 34 controls. Out of the 52 scanned adolescence ADHD participants, 3 participants were excluded due to one having developed comorbid bipolar disorder, another schizophrenia and another a clinically significant head injury (a skull fracture) by the time of the study. The final study size for the structural MRI was 49 young adult participants with adolescence ADHD.

The exclusion criteria used for both groups were mental retardation according to the Finnish Hospital Discharge Register or previous studies on this cohort; autism-spectrum disorders; speech development disorder with evident cognitive sequelae; psychotic disorder, or serious neurological conditions such as epilepsy, serious head injury or multiple sclerosis.

### Psychiatric assessment

On the day of the MRI scan, participants were evaluated with a clinical assessment, including urine drug screen, SCID (Structured Clinical Interview for DSM-IV) and, in addition, an in-house developed interview to rate the severity of ADHD symptoms in detail. This interview used both DSM-IV and ICD-10 ADHD criteria, allowing scoring on a list of 9 attention, 5 hyperactivity and 4 impulsivity symptoms. Diagnostic assessment was made according to DSM-IV criteria. Clinical answers to the incidence of the 9 inattentive, 5 hyperactivity and 4 impulsivity symptoms were labelled according to frequency of the symptoms and were defined as never/rarely, sometimes, often and very often. Scores ranging from 0 to 3 were attached to this frequency scale. Any score of two or three (often or very often) was deemed to be a positive clinical correlate of that symptom.

### Structural MRI data acquisition and preprocessing

All participants were scanned using GE Signa EchoSpeed HDx 1.5 Tesla MRI scanner in Oulu University Hospital. T1-weighted images were acquired with inversion recovery (IR)-prepared (“BRAVO”) 3D Fast Spoiled Gradient Echo (FSPGR) sequence using the following parameters: TR 12.4 ms, TE 5.2 ms, FA of 20°, FOV 24 cm × 24 cm, 256 × 256 acquisition matrix, 1 mm slice thickness, half k-space coverage in the phase encoding direction (GE “fractional NEX” with 0.5 factor). Structural data were analysed with FSL-VBM, a voxel-based morphometry style pipeline analysis [32–40]. We used the default FSL pipeline, including non-linear registration, and modulation, and applying a smoothing kernel of 3 mm.

### Group-level statistical analysis of brain structure

For the between-group comparisons, between-group statistics were performed using a permutation-based inference tool for nonparametric statistical thresholding in FSL’s randomise Monte Carlo permutation toolkit using 5000 permutations [41], with age at scan, handedness and sex as covariates. The significance threshold for between-group and time differences was set at *p* < 0.05, family-wise error corrected for multiple comparisons across voxels using the threshold-free cluster-enhancement (TFCE) option in the randomise permutation-testing tool in FSL, which results in fundamentally voxelwise statistics whilst also taking into consideration the size of clusters of significant contiguous groups of voxels. To help interpret the importance of any structural deficits, we planned to utilise the group difference results of the structural analysis to create a region of interest in which we could examine whether there were also functional brain imaging abnormalities in the adolescence ADHD group (see below).

### Functional MRI data acquisition

A random sub-sample of approximately two-thirds of the individuals participating in the structural scans also undertook fMRI scans on the same day using the same scanner as the structural scan using an EPI GRE sequence (TR = 1800 ms, TE = 40 ms, FOV = 25.6 cm × 25.6 cm, matrix 64 × 64, flip angle 90°, 28 oblique axial slices, slice thickness 4 mm, inter-slice gap = 0.4 mm, 344 volumes per run). We ran this sequence in the two separate runs. The first 4 volumes were removed from each run because of T1 equilibrium effects. Finite resources prevented us from conducting fMRI on all participants.

### Sternberg working memory paradigm

We employed a Sternberg task of working memory [42] during the fMRI scan; participants are instructed to focus on a point on the screen where a set of letters appear and stay on the screen 4 s. Next, a variable delay (of between 2 and 12 s) occurs, followed by a new letter (the probe) that may or may not have appeared in the letter set. The participant is instructed to press one button to denote if the probe letter did appear in the letter set and a different button if the probe letter is new, representing yes or no answers. An inter-trial interval with a jittered length of 3–5 s follows before the next trial begins with a new letter set (Fig. 3).

There were four different levels of difficulty, corresponding to four different loads of three, four, five and six letters, interspersed pseudo-randomly across the entire task. The task was divided in two runs, each with thirty-six trials, given a total of seventy-two trials for the whole task. The total duration per run was 10.3 min.

We excluded participants with low levels of accuracy from the fMRI analysis to ensure that the subjects whom we did analyse were genuinely and successfully utilising working memory. We applied an accuracy rate that required an accuracy rate of at least 75 % correct trials [43], so any person with an accuracy score below 54 (out of a possible 72) was excluded from the fMRI analyses.

### Functional MRI analysis

Functional analyses were performed using the “Analysis Group at the Oxford Centre for Functional MRI of the Brain” (FMRIB) software library (FSL) tools [34, 35, 44–46]. Skulls were first stripped automatically from each structural scan using FSL’s Brain Extraction Tool (BET) [35]. Following this, each individual’s functional scan’s brain was extracted using BET and was subsequently motion corrected (Motion Correction using FMRIB’s Linear Image Registration Tool, MCFLIRT) [38] as well as registered to its corresponding structural image using rigid body transformations and 7 degrees of freedom. After this, the corresponding scan was registered to the MNI standard brain using linear transformation with 12 degrees of freedom.

FSL’s FMRI expert analysis tool (FEAT) [34] was used to perform individual preprocessing and statistical analysis of each participant’s individual FMRI scan. High-pass temporal filtering of 100 s was applied to the FMRI images, which were then spatially smoothed using a Gaussian smoothing kernel of 6 mm. Intrinsic autocorrelations were modelled using pre-whitening. Regressors were convolved using a gamma (γ) hemodynamic response function. In the subject-level analysis, we used four regressors of interest (one for each working memory load: 3, 4, 5 or 6), with event onset times set at the start of each trial and duration set to the length of each trial (spanning encoding, maintenance and retrieval periods). We employed a regressor spanning these various periods of working memory function as we were more interested in obtaining a robust global measure of working memory function rather than breaking this process down into constituent parts. We used the “featquery” tool in FSL to extract the mean % signal change associated with each regressor within a region of interest defined by the results of the structural group difference analysis. Featquery uses a standard space-defined region of interest and maps that back into native space of each subject to then calculate the mean percent signal change associated with a given regressor for that ROI. Having extracted the mean ROI signal for each regressor of interest for each subject, we exported these to the statistics software package SPSS (version 21) to plot load-dependent activation and perform within and between-group tests (linear contrasts across load and group by linear trend interaction).

## Results

### Demographics and clinical outcomes: ADHD status in young adulthood

Demographics are detailed in Tables 1 and 2. There were 5 (10.2 %) participants diagnosed with ADHD during adolescence that after the clinical interview were deemed to still meet DSM-IV ADHD criteria. Of these participants, there were 2 participants whose subtype was defined as inattentive and 3 participants who were deemed to have the combined ADHD subtype. The scores for the ADHD group for the inattentive symptoms were 4.5 (SD 5.9), 2.7 (SD 3.6) for the hyperactivity symptoms and 1.8 (SD 2.2) for the impulsivity symptoms. In controls, the scores for the inattentive, hyperactivity and impulsivity symptoms were 1.1 (SD 1.9), 0.8 (SD 1.4) and 0.5 (SD 0.9). One ADHD participant was receiving treatment with stimulant ADHD medication at the time of the scan. Of the participants who no longer met ADHD criteria, the mean number of DSM-IV symptoms (i.e. domains meeting threshold of at least “often”) was 2 (range 0–9).

**Table 1:** Demographic description of the groups for the structural MRI

		Adolescence ADHD^a^ (*n* = 49)	Controls (*n* = 34)	Total (*n* = 83)
Age		22.23 (SD 0.7)	22.95 (SD 0.4)	22.53 (SD 0.67)
Sex		37 M:12 F	17 M:17 F	54 M:29 F
Handedness		41 R:8 L	32 R:2 L	73 R:10 L
IQ	Mean (std dev)	96.6 (21.8)	112.2 (22.6)	103 (23.1)
Education	High school enrolment	37 (75.5 %)	8 (23.5 %)	45 (54.2 %)
	High school graduation	12 (24.5 %)	26 (76.5 %)	38 (45.7 %)
GAF current	Mean (std dev)	74.6 (15.5)	87.26 (4.5)	79.8 (13.7)
Drug use detected by urine sample	Amphetamine and other stimulants	0	0	0
	Benzodiazepine	0	0	0
	Buprenorphine	0	0	0
	Cannabis	5 (10.2 %)	1 (2.9 %)	6 (7.2 %)
	Cocaine	0	0	0
	Opioids	0	0	0

^a^Adolescence diagnosis of ADHD. *GAF* global assessment of function score

**Table 2:** Demographic description of the groups for the fMRI

		Adolescence ADHD^a^ (*n* = 21)	Controls (*n* = 23)	Total (*n* = 44)
Age		22.2 (SD 0.7)	23 (SD 0.4)	22.6 (SD 0.7)
Sex		16 M:5 F	13 M:10 F	29 M:15 F
Handedness		18 R:3 L	22 R:1 L	40 R:6 L
IQ	Mean (std dev)	96.4 (19.8)	111.09 (25.09)	102.5 (22.6)
Education	High school enrolment	17 (80.9 %)	5 (21.7 %)	22 (50 %)
	High school graduation	4 (19.1 %)	18 (78.3 %)	22 (50 %)
GAF current	Mean (std dev)	77.1 (11.2)	87.1 (4.47)	79.5 (13.8)
Drug use detected by urine sample	Amphetamine and other stimulants	0	0	0
	Benzodiazepine	0	0	0
	Buprenorphine	0	0	0
	Cannabis	2 (9.5 %)	1 (4.3 %)	3 (6.8 %)
	Cocaine	0	0	0
	Opioids	0	0	0

^a^Adolescence diagnosis of ADHD. *GAF* Global Assessment of Function score

### Brain structural group differences

**Fig. 1:**
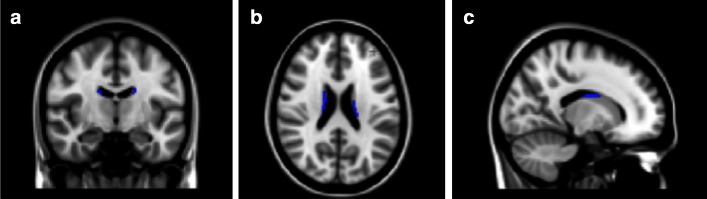
Adolescence ADHD individuals exhibit less grey matter volume (GMV) in bilateral caudate nucleus than controls (voxels significantly lower in GMV in the adolescence ADHD group, *p* < 0.05 family-wise error corrected across the whole brain, are shown in blue). The *left side* of the image is the *right side* of the brain in panels **a** and **b**. MNI co-ordinates:** a** y = −10;** b** z = 22;** c** x = 16

**Fig. 2:**
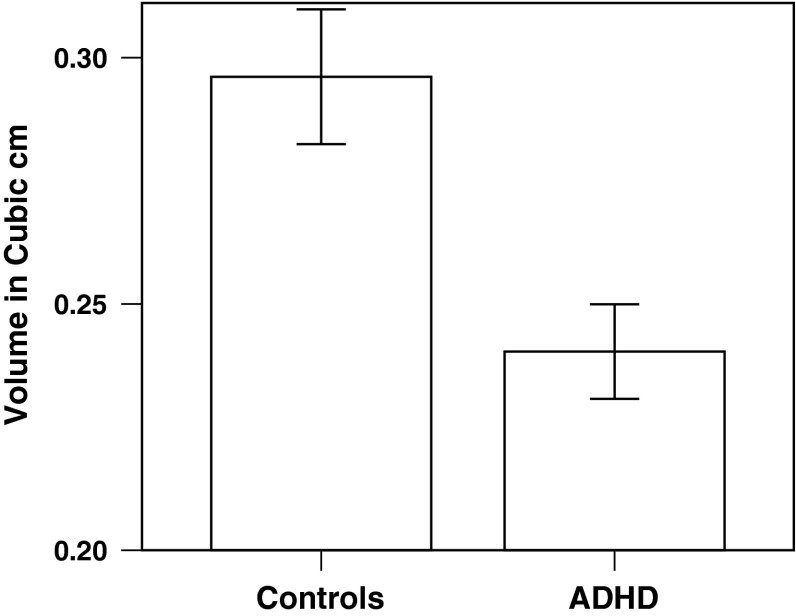
Plot of combined volume of the two caudate clusters depicted in Fig. 1 that contain voxels of reduced grey matter volume in adolescence ADHD compared to controls. Error bars are 95 % confidence intervals

There were significant differences in two clusters located in the left and right caudate, respectively, that had lower grey matter volume in the adolescence ADHD group when compared to controls (*p* < 0.05 FWE corrected, controlled for age at time of scan, sex, and handedness; Figs. 1, 2). The cluster located in the left caudate had its peak at MNI −18, −40, 22 (25 voxels); the cluster that was located in the right caudate had its peak at MNI 16, −32, 42 (38 voxels).

### Working memory behavioural performance results

There were 11 participants out of 32 who scored below 75 % of the total score in the adolescence ADHD group and one subject out of 24 in the control group; these participants were all excluded from the fMRI analysis. The total score for the remaining adolescence ADHD group (*n* = 21) was 62.76 (SD 4.43) and for the control group (*n* = 23) it was 66.87 (SD 2.32) (F = 5.59; df = 1.39; *p* = 0.023; age, IQ and sex used as covariates), showing that even when only considering participants who were engaging in the task largely successfully, the adolescence ADHD group performed worse than controls. There were very few missing answers, with no differences between groups; the ADHD group had on average 0.67 (SD 1.06) missing answers and the controls had 0.17 (SD 0.49), *p* = 0.19.

### fMRI results

**Fig. 3:**
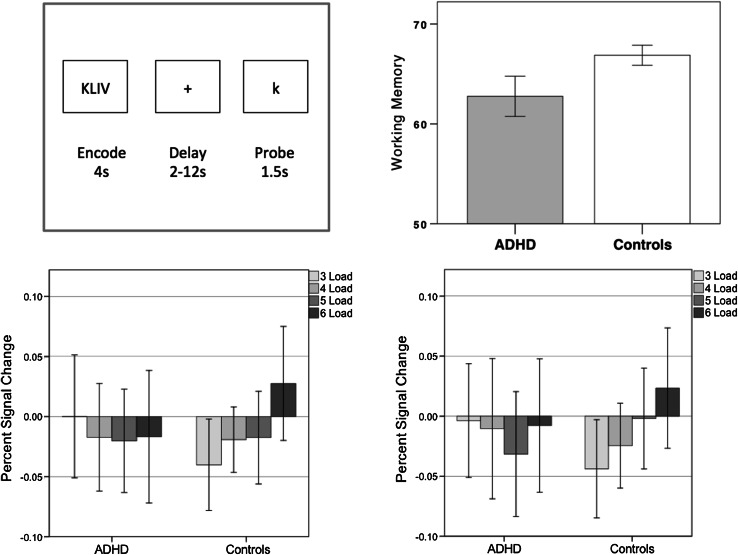
*Upper left panel* task design. Participants are shown a set of 3, 4, 5, or 6 letters, followed by a variable delay then a probe letter that may or may not have appeared in the letter set; the task is to indicate whether or not the probe appeared in the letter set or not. *Upper right panel* adolescence ADHD participants scored significantly lower on the task than control participants (F = 5.59; df = 1.39; *p* = 0.023); error bars are 95 % confidence intervals. *Lower panels* mean fMRI percent signal change within the left (*lower left panel*) and right (*lower right panel*) caudate ROIs defined in Fig. 1. Error bars are 95 % confidence intervals. The controls show significant trends of increasing activation with increasing memory load bilaterally (*p* < 0.05); this trend is not present in adolescence ADHD participants (*p* > 0.5)

We used the left and right caudate regions in which we had observed structural brain differences as masks in which to investigate functional group differences. For each run, we extracted the percent signal change for each load from each participant with FSL-featquery; for each load we then, for each participant, averaged across the two runs and plotted the group differences (Fig. 3). This indicated that in both ROIs, the control group exhibited clear load-dependent activation, with higher activity at high working memory loads (confirmed by within group repeated measures ANOVA, linear contrast across loads: left caudate, F = 4.44, df = 1.22, *p* = 0.047; right caudate F = 4.45, df = 1,22, *p* = 0.047); the ADHD group, however, failed to showed increased activation at higher working memory loads (within group repeated measures ANOVA, linear contrast across loads: left caudate, F = 0.22, df = 1, 20, *p* = 0.64; right caudate, F = 0.10, df = 1, 20, *p* = 0.76). In both left and right ROIs, there was a group difference of marginal significance in the strength of the linear association across loads (group by linear contrast interaction: left caudate F = 3.02, df = 1, 42, *p* = 0.09: right caudate: F = 2.91, df = 1, 42, *p* = 0.095). A summary measure of right caudate load-dependent activation (load 6 percent signal change minus load 3 percent signal change) predicted memory performance in controls (rho = 0.44, *p* = 0.03) but not in patients (rho = -0.03, *p* = 0.88).

### Results comparing those who continued to meet ADHD diagnostic criteria versus those who did not

To assess whether the group difference was driven by large abnormalities in the small group who continued to meet diagnostic criteria, we compare memory performance and brain structural and functional measures in the current ADHD versus former ADHD participants. There were no differences in memory score (current ADHD mean score 63.0, SD 5.3; former ADHD mean score 57.1, SD 9.0, *t* = 1.1, df = 29, *p* *=* 0.3); or in left caudate volume (current 0.08, SD = 0.01; former 0.08, SD = 0.1) *t* = 0.44, df = 47, *p* = 0.66) or in right caudate volume (current 0.15, SD = 0.02; former 0.16 SD = 0.02; t = 0.77, df = 47, *p* = 0.44); and there were no differences in fMRI caudate measures: left caudate load-dependent activation current ADHD mean score 0.08, SD 0.07; former mean score −0.03 SD 0.18, *t* = 1.0, df = 19, *p* = 0.34; right caudate activation current ADHD mean score 0.05, SD 0.07; former mean score −0.01, SD = 0.18; *t* = 0.58, df = 19, *p* = 0.57).

### Relationship between caudate MRI measures and clinical measures

Within the adolescence ADHD sample, there were no associations between current number of symptoms and brain structural measures (left caudate volume *r* = 0.06, *p* = 0.71; right caudate volume *r* = −0.11, *p* = 0.47) or functional measures (left caudate activation *r* = 0.2, *p* = 0.43; right caudate activation *r* = 0.35, *p* = 0.16). There were no associations between current GAF and brain structural (left caudate volume *r* = −1.88, *p* = 0.20; right caudate volume *r* = −0.14, *p* = 0.37) or functional measures (left caudate activation *r* = 0.81, *p* = 0.73; right caudate activation *r* = 0.87, *p* = 0.71). There were no associations between the number of symptoms in adolescence and the adult structural or functional measures (left caudate volume *r* = −0.12, *p* = 0.40; right caudate volume *r* = −0.13, *p* = 0.39; left caudate function *r* = 0.06, *p* = 0.80; right caudate function *r* = 0.13, *p* = 0.59).

## Discussion

### Adult ADHD assessment outcomes

The majority of young adults who had been diagnosed with current definite ADHD in adolescence no longer met DSM-IV diagnostic criteria for ADHD by the age of 20–24. The percentage of adolescents who have a persistent diagnosis into adulthood varies across centres and, necessarily, according to current diagnostic criteria. The exact diagnostic criteria for ADHD continue to evolve, and the 2013 revision of the DSM (DSM-5) recognised that adults require less symptoms to meet diagnostic criteria than children. The percentage of patients who continued to meet DSM-IV diagnostic criteria in our study is similar to the meta-analysis of Faraone and colleagues [47], but smaller than some clinical follow-up samples (e.g. 48). This may reflect the way that our participants were originally identified and diagnosed. The NFBC86 adolescence ADHD sample, from which our sample is drawn, was diagnosed in a general population screening procedure, with less than one-third having the “combined” subtype at age 16 [26], and is likely to be a slightly healthier group than those enrolled in many other studies who tend to be comprised mainly of patients with the combined subtype who attend specialist psychiatric services for ADHD treatment. The emphasis in the current report is not whether participants diagnosed with ADHD in adolescence continue to meet diagnostic criteria or not, but rather whether or not brain structure and function are normal or not in adulthood [18, 19].

### Brain structure

The structural abnormalities we found provide strong evidence that the caudate is abnormal in ADHD. Previously there has been inconsistent evidence of the structural deficits observed in the caudate for non-medicated people with ADHD. While some groups have established a decrease in caudate volume in children with ADHD [8, 11] other groups have, contrary to this, found higher caudate volumes which have been linked to exposure to medication [49]. Our study confirms evidence of grey matter volume deficits found in the caudate of adults who were diagnosed with ADHD during in childhood or adolescence [18]. However, in the only previous study to examine adult brain structural follow-up, 97 % of participants had taken methylphenidate medication, and our study shows that caudate differences were not ADHD medication induced. Although less volume is generally considered to be disadvantageous when interpreting VBM studies, strictly speaking, it is not necessarily detrimental, and we therefore elected to utilise our structural areas of deficits as a mask in which to assay, using a working memory fMRI task, whether caudate function was normal or abnormal in the adolescence ADHD group. The analysis showed that the caudate areas in which we identified structural deficits in the adolescence ADHD group also manifested functional deficits.

### Working memory function and brain activation

Working memory impairments were present in adulthood in people diagnosed with ADHD in adolescence, even if formal diagnostic criteria are no longer met. The group differences we observed in working memory were not simply secondary to the ADHD group failing to engage in the task, as even when we excluded poor performers and limited to the analysis to participants who were scoring correct answers on the vast majority of trials, correct answers were still significantly less than in controls.

Consistent with the working memory performance abnormalities observed in adults diagnosed with ADHD in adolescence, we also showed that caudate activation during task performance was abnormal. Whilst we found load-dependent working memory activation in both left and right caudate in controls, there was no load-dependent activation in the adolescence ADHD group. Load-dependent activation in the right caudate predicted working memory performance in controls but not in patients.

The caudate has long been implicated in a working memory network that supplies support functions related to the prefrontal cortex [50, 51]. Specific caudate abnormalities have previously been found in visual working memory in children with ADHD [52] and our results extend these to show abnormalities in verbal working memory in adults diagnosed with ADHD in adolescence. Studies using a visual selective attention task have shown that children with ADHD tend to show a pattern of neural activity with less activation in fronto-striatal regions and most notably the body of the caudate [53]. Our findings indicate that in adulthood, people diagnosed with ADHD in adolescence show impairments in working memory function, with a bilateral failure to activate the caudate increasingly with higher working memory loads: this may contribute to the memory deficits in ADHD.

### Strengths and limitations

There are aspects of our work that are both strengths and limitations: as the participants were recruited from the general population they will differ in certain respects from patients with severe ADHD recruited from specialist clinics. As both the adolescence ADHD group and controls were drawn from the same population-based birth cohort, they are matched in place of birth, and age, and will have been exposed to many similar environmental influences. Our control group was representative of the general population in being balanced in representation from males and females, whereas adolescence ADHD participants were mainly male. However, group differences in brain structure and cognitive performance persisted after controlling for gender. Our study shows that there are important residual deficits in young adults diagnosed with ADHD during adolescence in caudate volume, in working memory performance and associated caudate functional activity. The current work also benefits from a population sample that is, save for one participant, ADHD stimulant medication naïve, proving that the results are not driven by stimulant medication treatment: we are not aware of any similar studies previously published. The sample size of 83 participants (49 with an adolescence diagnosis of ADHD) is modest by the standards of epidemiology and some other research methods; however, a previous study with a smaller sample size (12 patients and 12 controls) was able to demonstrate that medicated patients with ADHD in childhood that persisted to adulthood had widespread fronto-parietal deficits as measured with fMRI during response inhibition [54]. We did not demonstrate any straightforward linear associations within the ADHD group between MRI measures and clinical measures. However, this is perhaps not unsurprising given that we noted that the “normal” relationship between caudate function and memory performance that we observed in controls was not present in adolescence ADHD participants.

## Conclusions

Although people diagnosed with ADHD in adolescence may recover clinically sufficiently to no longer meet diagnostic criteria, they may continue to manifest abnormalities in caudate structure and function and working memory performance in early adulthood. The results emphasize the importance of taking a wider perspective on ADHD outcomes than simply whether or not a particular patient meets diagnostic criteria at any given point in time.
